# SMLS-YOLO: an extremely lightweight pathological myopia instance segmentation method

**DOI:** 10.3389/fnins.2024.1471089

**Published:** 2024-09-25

**Authors:** Hanfei Xie, Baoxi Yuan, Chengyu Hu, Yujie Gao, Feng Wang, Yuqian Wang, Chunlan Wang, Peng Chu

**Affiliations:** ^1^School of Electronic Information, Xijing University, Xi’an, China; ^2^Xi’an Key Laboratory of High Precision Industrial Intelligent Vision Measurement Technology, Xijing University, Xi’an, China; ^3^Graduate Office, Xijing University, Xi’an, China

**Keywords:** pathological myopia, SMLS-YOLO, instance segmentation, lightweight, image feature extraction

## Abstract

Pathological myopia is a major cause of blindness among people under 50 years old and can result in severe vision loss in extreme cases. Currently, its detection primarily relies on manual methods, which are slow and heavily dependent on the expertise of physicians, making them impractical for large-scale screening. To tackle these challenges, we propose SMLS-YOLO, an instance segmentation method based on YOLOv8n-seg. Designed for efficiency in large-scale screenings, SMLS-YOLO employs an extremely lightweight model. First, StarNet is introduced as the backbone of SMLS-YOLO to extract image features. Subsequently, the StarBlock from StarNet is utilized to enhance the C2f, resulting in the creation of the C2f-Star feature extraction module. Furthermore, shared convolution and scale reduction strategies are employed to optimize the segmentation head for a more lightweight design. Lastly, the model incorporates the Multi-Head Self-Attention (MHSA) mechanism following the backbone to further refine the feature extraction process. Experimental results on the pathological myopia dataset demonstrate that SMLS-YOLO outperforms the baseline YOLOv8n-seg by reducing model parameters by 46.9%, increasing Box mAP@0.5 by 2.4%, and enhancing Mask mAP@0.5 by 4%. Furthermore, when compared to other advanced instance segmentation and semantic segmentation algorithms, SMLS-YOLO also maintains a leading position, suggesting that SMLS-YOLO has promising applications in the segmentation of pathological myopia images.

## 1 Introduction

Myopia is a condition where the eye’s refractive system focuses external light in front of the retina, resulting in distant objects appearing blurry because they are focused before the retina (Baird et al., 2020). It is a major cause of vision impairment in humans (Modjtahedi et al., 2018). Currently, over 1.4 billion people worldwide suffer from myopia; of these, 160 million people suffer from high myopia. By 2050, it is projected that the number of people with myopia is expected to exceed 4.7 billion, and this trend is expected to continue to accelerate (Holden et al., 2016). The rapid increase in myopia has become a significant global public health concern (Dolgin, 2015). Moreover, the rising prevalence of high myopia has led to an increase in the incidence of pathologic myopia. Pathologic myopia is distinct form of myopia, often characterized by axial myopia that has advanced to the stage of myopic maculopathy. It is marked by features such as posterior staphyloma and various fundus lesions. Unlike regular myopia, which primarily involves refractive errors, pathologic myopia also encompasses a complex set of fundus complications. Patients with this condition display distinctive funduscopic abnormalities. It remains unclear whether pathologic myopia progresses in parallel with regular myopia (Ohno-Matsui et al., 2021). Research by scholars, including Xu et al. (2006), indicates that pathologic myopia has emerged as the primary of irreversible blindness and the second most common cause of low vision, surpassed only by cataracts. As a result, it has become a critical focus in the prevention and management of myopia. Ohno-Matsui et al. (2015) proposed a grading system for myopic maculopathy, categorizing it into five grades, including three additional lesions: lacquer cracks, choroidal neovascularization, and Fuchs spots. Based on this standard, a diagnosis of pathologic myopia can be established at grade 2 or higher, or in the presence of at least one of these additional lesion.

In recent years, the prevalence of myopia among children and adolescents in China has been steadily increasing, leading to a corresponding increase in the incidence of pathologic myopia. The latest survey data shows that the overall myopia rate among Chinese children and adolescents has reached 51.9%, with a noticeable trend toward younger ages (Myopia Prevention and Control Guidelines, 2024). In response to this trend, various provinces have proactively launched school-based myopia screening and prevention programs. These programs involve establishing refractive profiles for students, scheduling follow-up visits, and implementing comprehensive prevention and treatment strategies. Such measures include regular vision checks, increasing outdoor activity time, improving classroom lighting conditions, and promoting scientific eye care knowledge. All these efforts are aimed at reducing the prevalence of myopia and preventing the onset of pathologic myopia. However, implementing large-scale screening for pathologic myopia faces challenges. The detection of pathologic myopia heavily relies on the professional knowledge and experience of ophthalmologists, primarily through manual procedures. This reliance leads to low efficiency and high costs. Additionally, the scarcity of ophthalmologists makes it challenging to conduct large-scale screenings, limiting the reach of early diagnosis and treatment. Furthermore, current detection algorithms in practical applications suffer from insufficient accuracy and high computational resource consumption, resulting in slow detection speeds and high misdiagnosis rates. These challenges hinder the efficiency and coverage of efforts to prevent and control pathological myopia.

To tackle these challenges, it is essential to develop more accurate, efficient, and resource-efficient auto detection technologies. This development demands advancements not only in the accuracy and efficiency of algorithms but also in the practical applications’ convenience and user-friendliness. By integrating advanced technologies like artificial intelligence and machine learning, we anticipate a significant enhancement in the precision and efficiency of pathological myopia detection. These innovations aim to reduce detection costs and broaden screening coverage, ultimately benefiting a larger patient population.

In recent years, with the advancement of fundus photography and Optical Coherence Tomography (OCT) technologies, doctors have been able to acquire patients’ ocular data more conveniently, non-invasively, and visually (Li, 2023). This progress has facilitated the widespread application of image recognition-based diagnostic methods for pathological myopia. Concurrently, the rapid development of Artificial Intelligence (AI) has demonstrated extraordinary potential across various industries. As a significant branch of AI, deep learning has shown immense promise in the automated analysis of medical information and imaging. In the field of ophthalmology, where the diagnosis of many diseases relies on ocular imaging, AI-assisted image recognition technology has been extensively applied in the diagnosis of a variety of eye conditions, including diabetic retinopathy, age-related macular degeneration, and glaucoma.

In the early stages, the complexity of annotating pathological myopia lesion areas led to difficulties in annotation, resulting in a scarcity of datasets for pathological myopia segmentation. This also led to early deep learning-based research on pathological myopia focusing primarily on the classification of pathological myopia images. In 2021, Rauf and colleagues proposed a machine learning-based algorithm for the identification of pathological myopia. They first pre-processed the pathological myopia and then input it into a CNN (Convolutional Neural Network) for identification, achieving an AUC (Area Under the Curve) score of 0.9845 (Rauf et al., 2021). Lu and others used the ResNet50 classification network for the classification of pathological myopia images, achieving an accuracy rate of 97.08% (Lu et al., 2021). Qin and colleagues proposed a CNN-based screening system for pathological myopia, which achieved an accuracy rate of 99.7% (Qin et al., 2023).

In the research of pathological myopia, although early work focused mainly on image recognition, the importance of segmentation has gradually become apparent as research has progressed. Compared to recognition, segmentation can accurately locate and separate the lesion areas, which has a more direct significance for the accurate diagnosis and treatment of pathological myopia. Through segmentation, not only can the morphology and changes of the lesion area be analyzed more meticulously, but it can also provide doctors with more detailed information about the lesions, helping to formulate more personalized and precise treatment plans. Therefore, segmentation technology has gradually taken a leading position in the automated analysis of pathological myopia, becoming a key link in achieving accurate diagnosis and intervention. However, real-time processing is an inevitable issue in large-scale screening scenarios. Although commonly used pixel-level semantic segmentation algorithms such as UNet (Ronneberger et al., 2015) and DeepLab V3 (Chen et al., 2018) perform well in accuracy, their processing speed is relatively slow, limiting the efficiency of AI-assisted diagnosis in large-scale screening, which restricts the work efficiency of AI-assisted diagnosis in large-scale surveys. Therefore, improving the speed and efficiency of algorithms is key to achieving broader screening and early intervention. In this context, instance segmentation technology has shown unique advantages. Compared to traditional pixel-level semantic segmentation, instance segmentation can not only accurately identify and segment each independent lesion area in the image but can also handle segmentation tasks for multiple types of lesions simultaneously. Through instance segmentation, the algorithm can more efficiently process complex fundus images, further improving the accuracy and speed of pathological myopia diagnosis.

In the current field of deep learning, instance segmentation is divided into single-stage and two-stage methods. Two-stage instance segmentation algorithms first use a detector to locate objects in the image, and then perform fine segmentation within each detected object area. The advantage of two-stage methods is that the segmentation results are usually more accurate because they can utilize the high-quality candidate areas provided by the detector. However, these methods typically have a large computational load and long inference times, making them less suitable for real-time applications. Single-stage instance segmentation algorithms, on the other hand, complete both detection and segmentation tasks within a single network, simplifying the process and increasing efficiency. Therefore, single-stage instance segmentation algorithms generally have higher detection speeds compared to two-stage methods and are more suitable for real-time detection. Single-stage instance segmentation algorithms, such as the YOLO-seg series, SOLO (Wang et al., 2022), and CenterMask (Lee and Park, 2020), segment targets directly in the image, combining efficiency and accuracy, making them suitable for real-time segmentation tasks. These algorithms are designed to balance the need for speed with the requirement for precision, which is particularly important in applications where rapid processing is crucial, such as in medical imaging for real-time diagnostics or in autonomous systems that require immediate environmental understanding.

In summary, considering the need for real-time performance in large-scale screening for pathologic myopia detection, single-stage instance segmentation algorithms are particularly suitable. These algorithms maintain high detection accuracy while offering faster processing speeds, meeting the real-time requirements of pathologic myopia detection. Therefore, this paper proposes a novel single-stage instance segmentation algorithm, SMLS-YOLO. This algorithm is specifically designed for the segmentation of lesion areas in fundus images of pathologic myopia, aiming to achieve efficient and accurate real-time segmentation to meet the demands of large-scale screening.

In SMLS-YOLO, extreme lightweight processing has been implemented to meet the real-time requirements of the algorithm, with the model’s parameter count being only 1.7 M, significantly smaller than other instance segmentation algorithms. First, StarNet (Ma et al., 2024) is introduced as the backbone to extract image features. Next, to better integrate the features extracted by the backbone, we propose an efficient feature extraction module, C2f-Star, which enhances the detection accuracy of the algorithm. Additionally, to better adapt to different lesion area sizes, we propose a segmentation head based on shared convolution. Using shared convolution significantly reduces the number of parameters. Alongside shared convolution, a scale layer is employed to adjust features, addressing the inconsistency in target scales segmented by each detection head. Finally, the MHSA (Han et al., 2023) attention mechanism is incorporated, greatly enhancing the model’s performance. Combining these features, SMLS-YOLO not only improves the speed and accuracy of detection and segmentation but also provides a practical solution, offering strong support for early diagnosis and effective intervention of pathologic myopia. By applying our algorithm, it is expected to significantly enhance the screening efficiency of pathologic myopia, meeting the urgent need for rapid and accurate detection in clinical and public health fields.

The main contributions of this paper include:

1. This paper proposes SMLS-YOLO, a real-time instance segmentation algorithm based on a single-stage approach. It is designed to meet the need for real-time detection in large-scale screenings for pathological myopia.
2. We propose a lightweight instance segmentation head called Segment_LS. The segmentation head in YOLOv8 accounts for 30.7% of the total network parameters. Segment_LS significantly cuts down the parameter count by utilizing shared convolutions and a scale layer to adjust features, addressing the challenge of inconsistent target scales detected by each detection head. This results in approximately a 75.6% reduction in the parameters of the segmentation head itself, and nearly halves the total number of model parameters.
3. An efficient feature extraction module, C2f-Star, is proposed, which is designed to reduce computational load and the number of parameters while enhancing the model’s performance.
4. The incorporation of the Multi-Head Self-Attention (MHSA) mechanism notably boosts the model’s performance.
5. Comprehensive experiments conducted on a pathological myopia dataset reveal that SMLS-YOLO exhibits exceptional detection capabilities even under extremely lightweight conditions.

The remainder of this paper is organized as follows: Section 2 reviews related work on pathologic myopia detection. Section 3 presents the proposed SMLS-YOLO and related improvement strategies. Section 4 provides implementation details. Section 5 analyzes the experimental results. Section 6 concludes the paper and discusses future research directions.

## 2 Relate work

### 2.1 Methods based on traditional image processing

In the early field of pathologic myopia instance segmentation, research primarily focused on the application of traditional image processing techniques. Initially, fine preprocessing of fundus images, including key techniques such as noise reduction filtering and contrast enhancement, was aimed at improving image quality. Following this, methods such as region-growing algorithms, threshold segmentation techniques, and K-means clustering analysis were used for the identification and segmentation of lesion areas. Specifically, Aquino et al. (2010) proposed a template-based segmentation method that integrated morphological analysis with edge detection techniques, successfully achieving approximate segmentation of the circular boundary of the optic disk. GeethaRamani and Dhanapackiam (2014) further explored the synergistic effect of template matching and morphological operations, making advancements in the accuracy of optic disk localization. Marín et al. (2015) combined morphological operations with efficient edge detection strategies to achieve precise localization of the fundus image center and detailed segmentation of the optic disk and retinal areas, providing new insights for analyzing complex fundus structures. Chakravarty and Sivaswamy (2017) proposed an innovative boundary conditional random field model that comprehensively considered the depth interactions and color gradient information of the optic disk and cup boundaries. By incorporating supervised depth estimation, this model achieved more accurate boundary extraction, offering a new method for detecting fundus lesions.

Although the aforementioned methods have demonstrated potential in lesion area detection to some extent, their sensitivity to image noise, adaptability to complex lesion morphologies, and generalization capabilities still require further improvement.

### 2.2 Methods based on deep learning

In recent years, with the rapid development of deep learning technology, especially the application of convolutional neural networks (CNN) in instance segmentation, it has gradually become a research hotspot. These methods leverage the powerful feature extraction capability and automated learning process of deep learning, significantly improving the accuracy and efficiency of image segmentation. Due to their wide application in fields such as medical imaging, autonomous driving, and security surveillance, CNN-based instance segmentation algorithms have increasingly attracted attention and research, becoming a significant force driving the advancement of image processing and computer vision technologies.

Viedma and other scholars have proposed the use of the instance segmentation algorithm Mask R-CNN for multi-level segmentation of retinal OCT (Optical Coherence Tomography) images. Compared to the traditional U-Net method, this approach not only achieves higher segmentation performance but also simplifies the extraction process of boundary positions, significantly reducing inference time (Viedma et al., 2022). Hung-Ju Chen and other scholars utilized the instance segmentation algorithm Mask R-CNN to achieve precise segmentation of the choroid in myopic eyes. In their study, they designed a deep learning-based segmentation method that successfully separated and identified the choroidal region through instance segmentation of ocular images (Chen et al., 2022). Almubarak and other scholars proposed a two-stage method for locating the optic nerve head and segmenting the optic disk/cup (Almubarak et al., 2020).

These studies demonstrate the versatility and effectiveness of instance segmentation algorithms in ophthalmic imaging, where precise localization and segmentation of different layers and structures are essential for accurate diagnosis and treatment planning. The adoption of advanced deep learning techniques like Mask R-CNN has the potential to revolutionize the field by providing more accurate and efficient tools for ophthalmologists.

## 3 Methods

### 3.1 SMLS-YOLO

**Figure 1:**
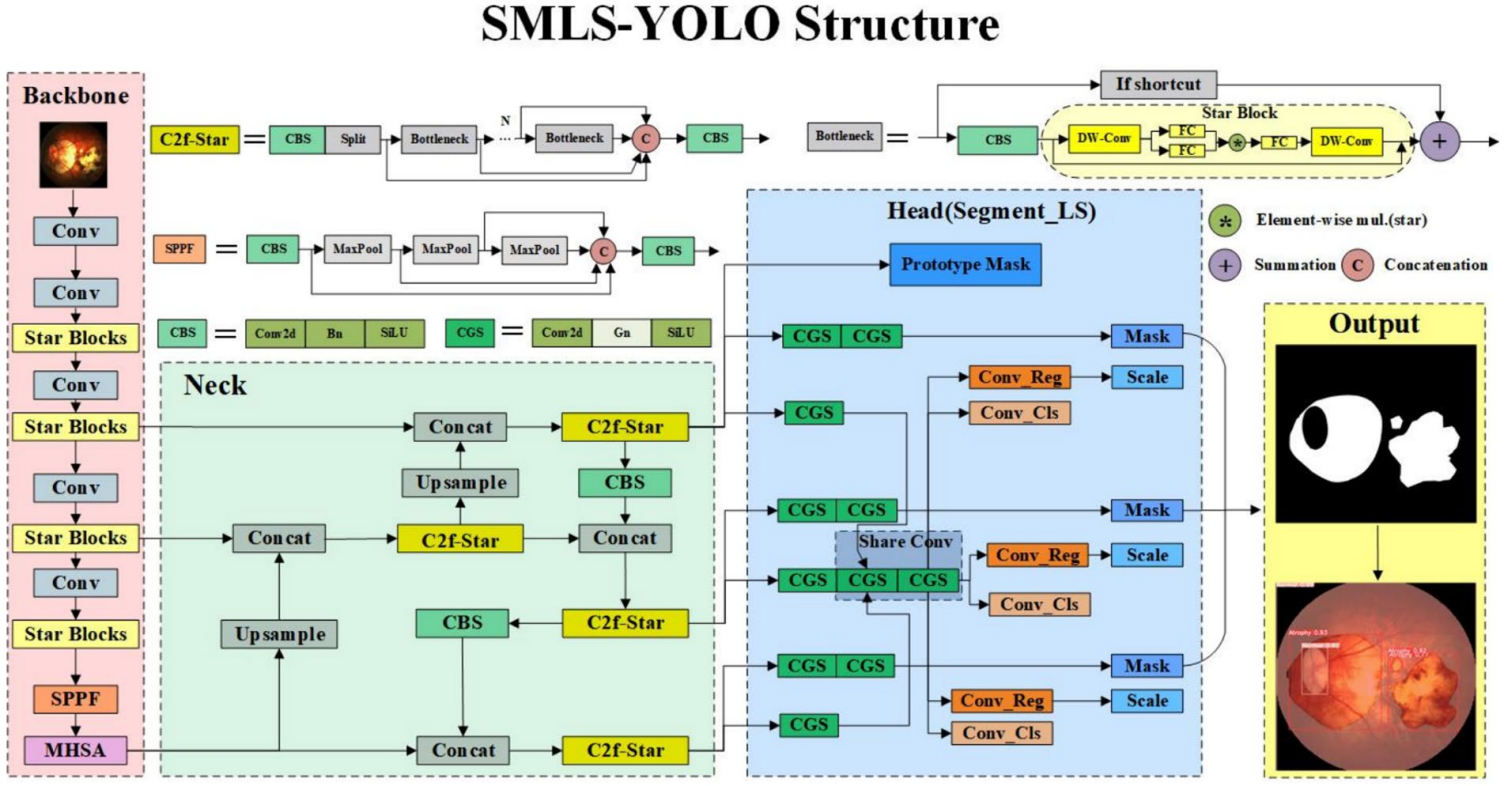
SMLS-YOLO network structure diagram.

In this paper, we propose an improved method based on YOLOv8 to achieve high-precision, rapid detection and instance segmentation of pathological myopia images, meeting the requirements of large-scale screening. The method, SMLS-YOLO, is specifically designed for the segmentation of lesion areas in fundus images of pathological myopia. Compared to the original YOLOv8, our SMLS-YOLO method has made significant improvements in the following four aspects:

1. Adopted a lightweight Backbone. To achieve extreme lightweighting, SMLS-YOLO employs StarNet as the model’s feature extraction network. This choice not only reduces computational resource consumption but also improves the model’s efficiency.
2. Proposed an efficient feature extraction module, C2f-Star. The innovative C2f-Star feature extraction module is introduced, which, while maintaining a lightweight model, better captures and extracts fine features in images, thereby improving segmentation accuracy.
3. MHSA attention mechanism. To enhance the model’s focus on lesion areas, we incorporated a multi-head self-attention mechanism (MHSA) into the model. MHSA effectively enhances the model’s performance in processing complex fundus images by focusing on key areas of the image, significantly improving segmentation accuracy.
4. Proposed a shared convolution-based segmentation head, Segment_LS. By using shared convolution, the number of parameters can be greatly reduced, making the model more lightweight. While using shared convolution, to address the issue of inconsistent target scales segmented by each detection head, a scale layer is used to scale the features. Figure 1 illustrates the network structure of SMLS-YOLO.

### 3.2 StarNet

**Figure 2:**
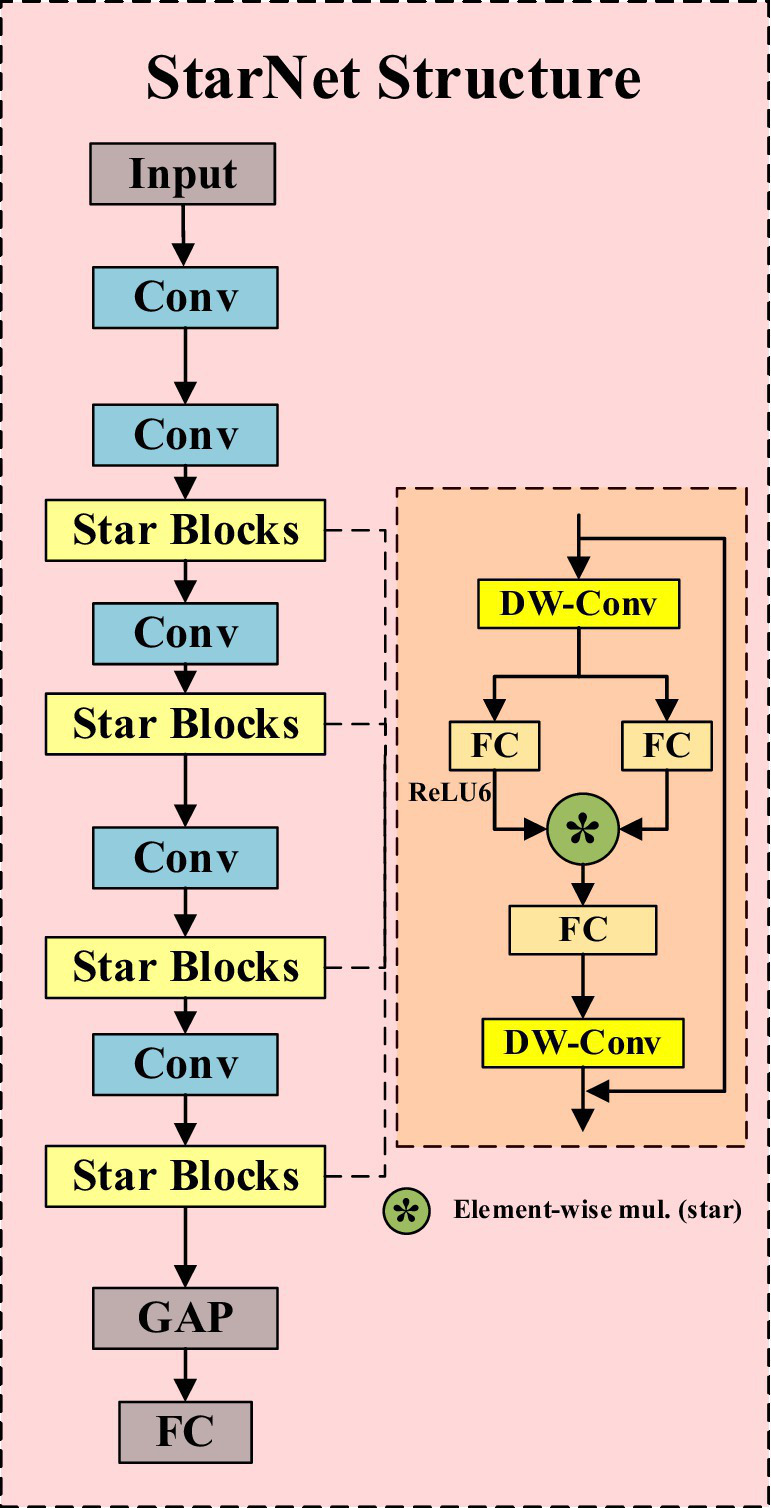
StarNet network structure diagram.

StarNet is an efficient convolutional neural network that not only inherits the strengths of traditional convolutional neural networks but also enhances the high-dimensionality and nonlinearity of feature representation through the innovative “star operation.” As shown in Figure 2, its structure mainly consists of convolutional layers and Star Blocks, with the latter integrating the “star operation.” The “star operation” maps image features into a high-dimensional nonlinear space through element-wise multiplication, significantly enhancing the expressive power of features without increasing the network’s width, thereby achieving efficient feature extraction and fusion.

The essence of StarNet lies in its ability to transform input features into an implicit high-dimensional feature space through simple element-wise multiplication. This mapping not only increases the dimensionality of the feature space but also enhances the network’s ability to express complex patterns without adding computational complexity. This characteristic allows StarNet to perform well while maintaining a compact network structure and efficient computation. In addition, StarNet not only has significant performance advantages but also maintains low latency under limited computational resources, making it suitable for real-time application scenarios. Incorporating StarNet as a feature extraction network brings many notable advantages. Firstly, the overall number of model parameters is significantly reduced, and the computational complexity is lowered, thereby accelerating the model’s inference speed. Secondly, StarNet’s efficient feature expression capability ensures the model’s accuracy.

### 3.3 C2f-Star

**Figure 3:**
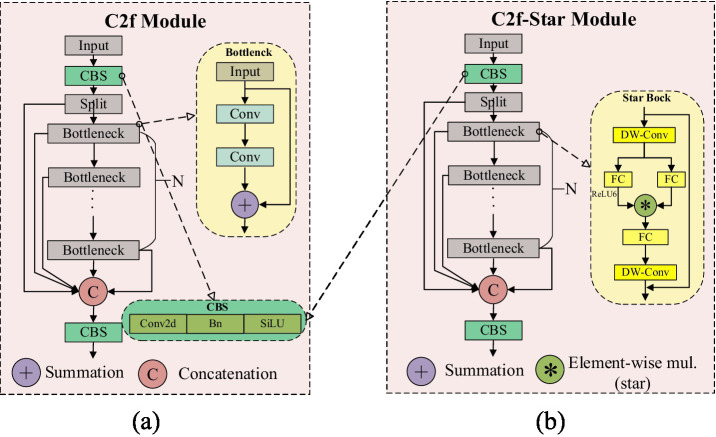
**(A)** C2f structure diagram. **(B)** C2f-star structure diagram.

In order to more effectively utilize the feature information extracted by the Backbone, we have integrated the “star operation” into the C2f module, proposing the C2f-Star module. Figures 3A,B respectively illustrate the structural diagrams of the C2f and C2f-Star modules. From the structural diagrams, it can be seen that the C2f-Star maintains the original basic structure of C2f while incorporating the “star operation” from StarNet to enhance the feature expression capability and the ability to capture complex patterns. Through this improvement, the C2f-Star module achieves a balance of efficiency and accuracy while maintaining a lightweight design.

In the design of the C2f-Star module, we have fully leveraged the advantages of the “star operation” in StarNet for feature extraction. StarNet generates high-dimensional features with rich expressiveness through the “star operation.” However, high-dimensional features alone are not sufficient to fully realize the potential of the entire network. Therefore, we have introduced the “star operation” into the C2f module to further optimize and process these features. The C2f-Star module not only inherits the advantages of StarNet’s “star operation” but also combines the efficient feature processing mechanism of the C2f module to provide more refined processing of these high-dimensional features. By integrating depthwise convolution and fully connected layers, the C2f-Star module not only retains the richness of the features but also further enhances the interaction between features, making the feature expression more accurate and effective.

### 3.4 Segment_LS segmentation head

The segmentation head of YOLOv8 adopts the method from YOLACT (Bolya et al., 2019), breaking down the instance segmentation task into two steps. YOLOv8 first generates a set of prototype masks, where each detection head outputs a set of coefficients for each instance target. These prototype masks are then weighted and combined to obtain the final instance segmentation result. However, the segmentation head of YOLOv8 has significant drawbacks. It uses shared prototype masks that are common to all instances. Although this approach is computationally efficient, it May fail to capture the detailed features of targets requiring fine features, resulting in less precise segmentation. Additionally, the global sharing nature of the prototype masks might overlook small targets or fail to precisely segment large targets, especially in densely populated scenes where instance masks May overlap, affecting segmentation accuracy. Due to these shortcomings, to maintain high precision, the segmentation head of YOLOv8 employs a large number of convolutional and feature extraction layers, leading to a large number of parameters. Practical tests show that the segmentation head of YOLOv8 accounts for 30.7% of the total network parameters.

**Figure 4:**
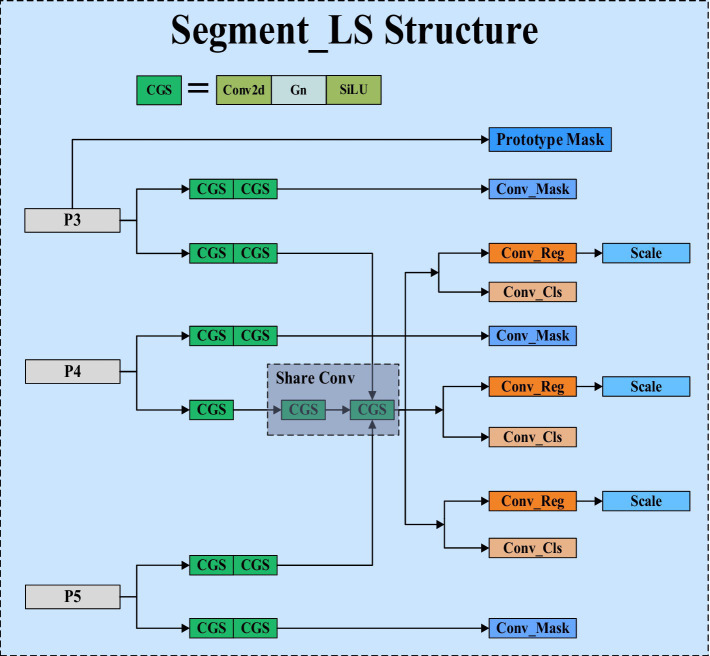
Segment_LS structure diagram.

In response to the aforementioned shortcomings, we have proposed a new type of efficient segmentation head called Segment_LS. Segment_LS no longer uses the shared prototype masks of the original YOLOv8, overcoming the inherent flaws of YOLOv8’s segmentation head. As a result, our segmentation head does not rely on a large number of parameters to improve accuracy, which significantly reduces the overall parameter count of the network. The structure of Segment_LS is shown in Figure 4.

In the design of the Segment_LS segmentation head, we first maintained the original Segment structure, allowing it to continue receiving feature maps from P3, P4, and P5 at different scales, thus preserving the segmentation head’s multi-scale feature fusion capability. Additionally, we introduced shared convolutions, GroupNorm, and Scale scaling operations into the Segment_LS segmentation head. Compared to BatchNorm, GroupNorm does not depend on batch size and performs particularly well in training with small batches or even single images. By incorporating GroupNorm into the segmentation head, detection and segmentation accuracy can be stably improved across various batch sizes. To address the issue of excessive computational load in the segmentation head, we introduced shared convolution layers in the paths of P4 and P5. This mechanism not only significantly reduces the model’s parameter count but also ensures consistent processing of features at different scales, enhancing the model’s information sharing capability across scales and thereby improving the model’s generalization ability and computational efficiency. To tackle the issue of inconsistent scales, we introduced a Scale layer for feature scaling alongside shared convolutions, ensuring that each detection head can perform object detection and segmentation at the optimal scale, resulting in more stable and accurate regression outcomes. This approach effectively mitigates the accuracy drop caused by inconsistent target scales. Furthermore, the Head part carries out tasks such as mask prediction (Conv_Mask), regression prediction (Conv_Reg), and classification prediction (Conv_Cls) through multiple parallel paths. This separated path design allows the model to optimize for each task specifically, avoiding interference between tasks, thereby enhancing overall performance. Lastly, to generate more precise initial features during image segmentation, we generate prototype masks through a separate path, providing a reliable foundational template for subsequent segmentation tasks. Ultimately, we conducted practical tests on the original segmentation head of YOLOv8 and the optimized segmentation head. The results showed that the optimized segmentation head has a parameter count of 0.25 M, with a total model parameter count of 1.7 M, accounting for 14.4% of the computational volume; in contrast, the original YOLOv8 segmentation head has a parameter count of 1.00 M, with a total model parameter count of 3.26 M, accounting for 30.7% of the computational volume. Through optimization, our segmentation head’s parameter count was reduced by approximately 75.6%, and the total model parameter count was nearly halved. This optimization significantly reduced the computational load while maintaining high accuracy.

### 3.5 MHSA attention mechanism

**Figure 5:**
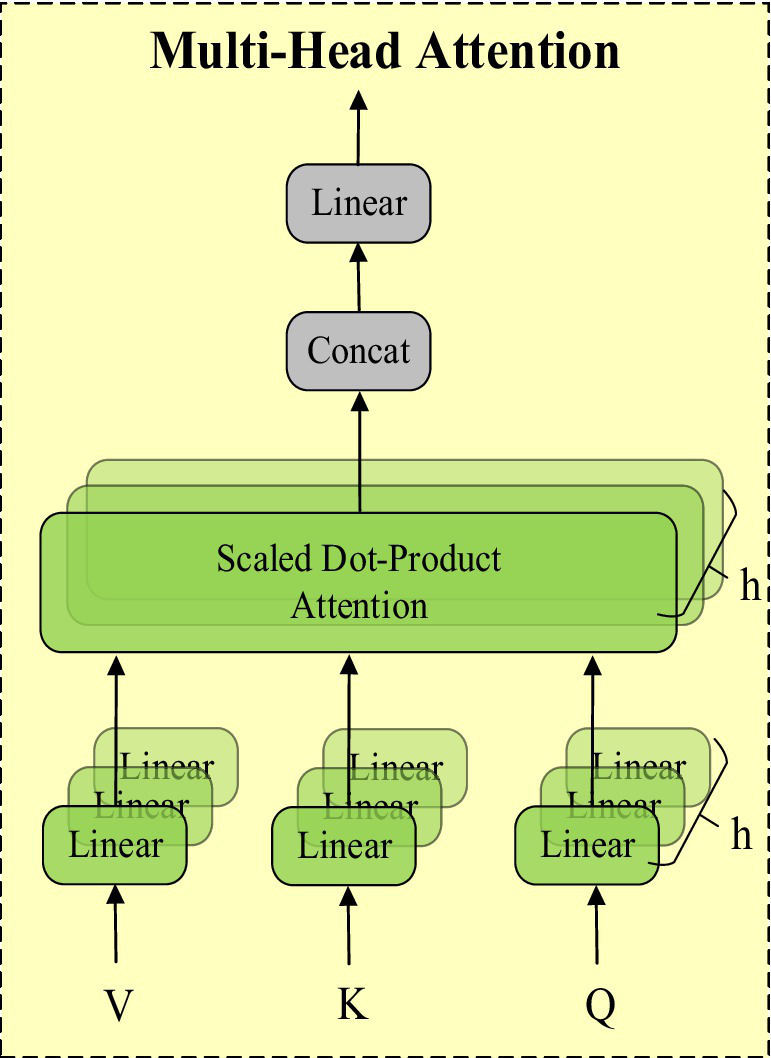
MHSA attention mechanism structure diagram.

The attention mechanism is an important technique in deep learning that enhances the model’s ability to focus on different parts of the input data. Its core idea is to assign differentiated weights to input features, enabling the model to focus more on features that contribute significantly to the task. The multi-head self-attention mechanism (MHSA) further extends this concept. MHSA calculates the correlations between input features by using multiple attention heads in parallel. Each attention head independently captures different feature relationships and then combines these results. This enhances the model’s feature representation capability and improves its ability to handle long-range dependencies. The structure of MHSA is shown in Figure 5.

MHSA processes input features in parallel through multiple attention heads, with each head independently calculating a set of attention weights and applying them to the features. These results are then concatenated and transformed linearly to generate the final output features. This allows MHSA to simultaneously focus on different parts of the input features, capturing richer inter-feature relationships.

We combined the characteristics of StarNet by introducing the multi-head self-attention mechanism (MHSA) after the Backbone to further enhance the model’s performance. StarNet’s “Star Operation” maps input features to a high-dimensional nonlinear feature space, enhancing expressive capability. MHSA captures long-range dependencies between features through parallel attention heads and integrates this information into feature representation. The combination of these two methods allows the model to capture local features and effectively integrate global features without increasing computational complexity, enhancing the richness and accuracy of overall feature representation.

## 4 Experiments

### 4.1 Experimental environment

All experiments covered in this paper were conducted on a deep learning workstation. The hardware configuration and experimental environment are shown in Table 1.

**Table 1:** Hardware configuration and experimental environment.

Name	Model
CPU	Intel Xeon Silver 4210
System	Windows 10
GPU	NVIDIA RTX 2080Ti 11GB
RAM	64 GB
Python	3.8.17
CUDA	11.6
Pytorch	1.8.0
Torchvision	0.9.0

Based on the above experimental conditions, we set the training epochs to 300, the batch size to 16, the initial learning rate to 0.01, the momentum to 0.937, the weight decay coefficient to 0.0005, the input image size to 640 × 640, and the number of workers to 8. We used YOLOv8’s mosaic data augmentation.

### 4.2 Evaluation metrics

Evaluation metrics are important tools for measuring model performance. The metrics used in this paper to evaluate model size include computational load (GFLOPS), number of parameters (Parameters), and frames per second (FPS). The metrics used to evaluate model accuracy include precision (P), average precision (AP) for each class, mean average precision (mAP) across all classes, and recall rate (R).

Precision is used to measure how many of the samples predicted as positive by the classification model are actually positive examples. The calculation formula is shown in Equation 1:


(1) $$P=\frac{TP}{TP+FP}$$

In the formula, *P* represents Precision, TP is the number of true positive cases, and FP is the number of false positive cases.

Recall rate (R) represents the proportion of correctly predicted positive samples to all actual positive samples. The calculation formula is shown in Equation 2:


(2) $$R=\frac{TP}{FP+FN}$$

In the formula, TP is the number of true positive cases, and FN is the number of false negative cases.

Average Precision (AP) is a commonly used evaluation metric to measure the accuracy of a model in information retrieval or object detection tasks across different classes or thresholds. It measures the model’s performance by calculating the area under the Precision-Recall curve. The calculation formula is shown in Equation 3:


(3) $$AP=\int P\left(R\right)dR$$

Mean Average Precision (mAP) is used to measure the accuracy of a model in information retrieval or object detection tasks across all classes or thresholds. The calculation formula is shown in Equation 4:


(4) $$\mathrm{mAP}=\frac{1}{N}\overset{N}{\underset{i=1}{\sum }}AP_{i}$$

In the formula, $AP_{i}$ represents the average precision for class i, and N represents the total number of classes.

To more intuitively demonstrate the training effect of the model, the mAP@0.5 comprehensive evaluation metric is introduced. mAP@0.5 represents the mAP when the IoU value is set to 0.5. When IoU > 0.5, it is considered that there is a predicted target within the predicted bounding box. When IoU < 0.5, it is considered that there is no predicted target within the predicted bounding box. mAP@0.5 can comprehensively evaluate the model’s localization and classification accuracy. The calculation formula is shown in Equation 5:


(5) $$\mathrm{mAP}@0.5=\frac{1}{N}\overset{N}{\underset{\begin{array}{c} i=1\\ IOU=0.5 \end{array}}{\sum }}AP_{i}$$

The F1-score is the best balance point that measures both precision and recall, providing a more comprehensive reflection of the model’s overall performance. The definition of the F1 score is shown in Equation 6:


(6) $$F1_{\mathrm{Score}}=2\times \frac{\mathrm{Precision}\times \mathrm{Recall}}{\mathrm{Precision}+\mathrm{Recall}}$$

FPS refers to the number of images the algorithm processes per second. The definition of FPS is shown in Equation 7:


(7) $$FPS=\frac{1}{T_{\mathrm{per}}}$$

where $T_{\mathrm{per}}$ represents the time taken by the algorithm to process a single fundus image.

Intersection over Union (IoU) represents the ratio of the intersection to the union between the predicted results and the ground truth, which can be used to assess the accuracy of segmentation models, as shown in Equation 8:


(8) $$IoU=\frac{TP}{TP+FP+FN}$$

TP represents the number of pixels in the lesion area of the fundus image that are correctly predicted, FP represents the number of pixels in the background area that are incorrectly predicted as being part of the lesion area in the fundus image, and FN represents the number of pixels in the lesion area of the fundus image that are incorrectly predicted as being part of the background area.

### 4.3 Dateset

**Figure 6:**
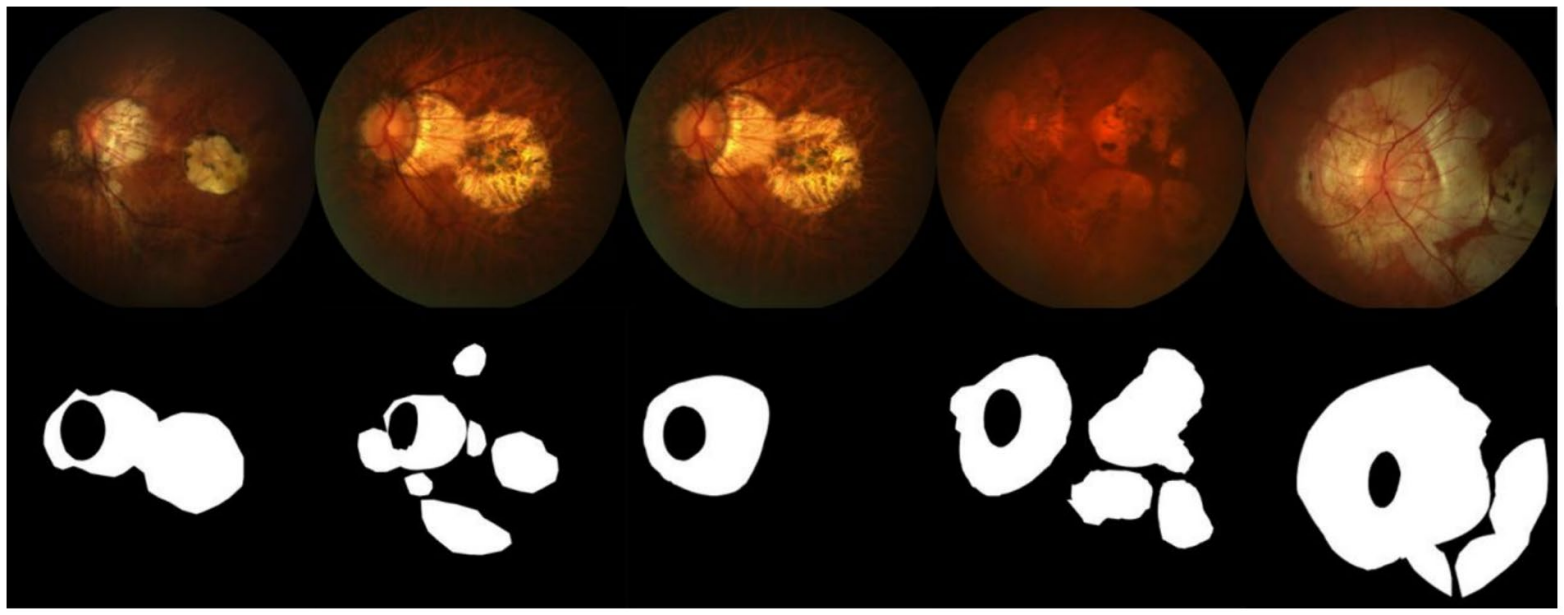
Sample images and masks from the dataset.

The dataset used in this paper is sourced from the PALM Pathological Myopia Lesion Detection and Segmentation Challenge, provided by the Zhongshan Ophthalmic Center of Sun Yat-sen University. The dataset includes 582 fundus images with annotations for atrophy and detachment lesions, and 213 fundus images without lesions. Each fundus image is annotated with typical lesions related to pathological myopia: patchy retinal atrophy (including peripapillary atrophy) and normal regions without lesions. Pixel-level lesion annotations were initially manually performed by seven ophthalmologists from the Zhongshan Ophthalmic Center, and the final gold standard annotation was created by another senior expert who integrated the results from the seven ophthalmologists. Additionally, the dataset contains 400 unannotated fundus images used as a test set. Some images from the dataset and their corresponding masks are shown in Figure 6.

### 4.4 Data augmentation

**Figure 7:**
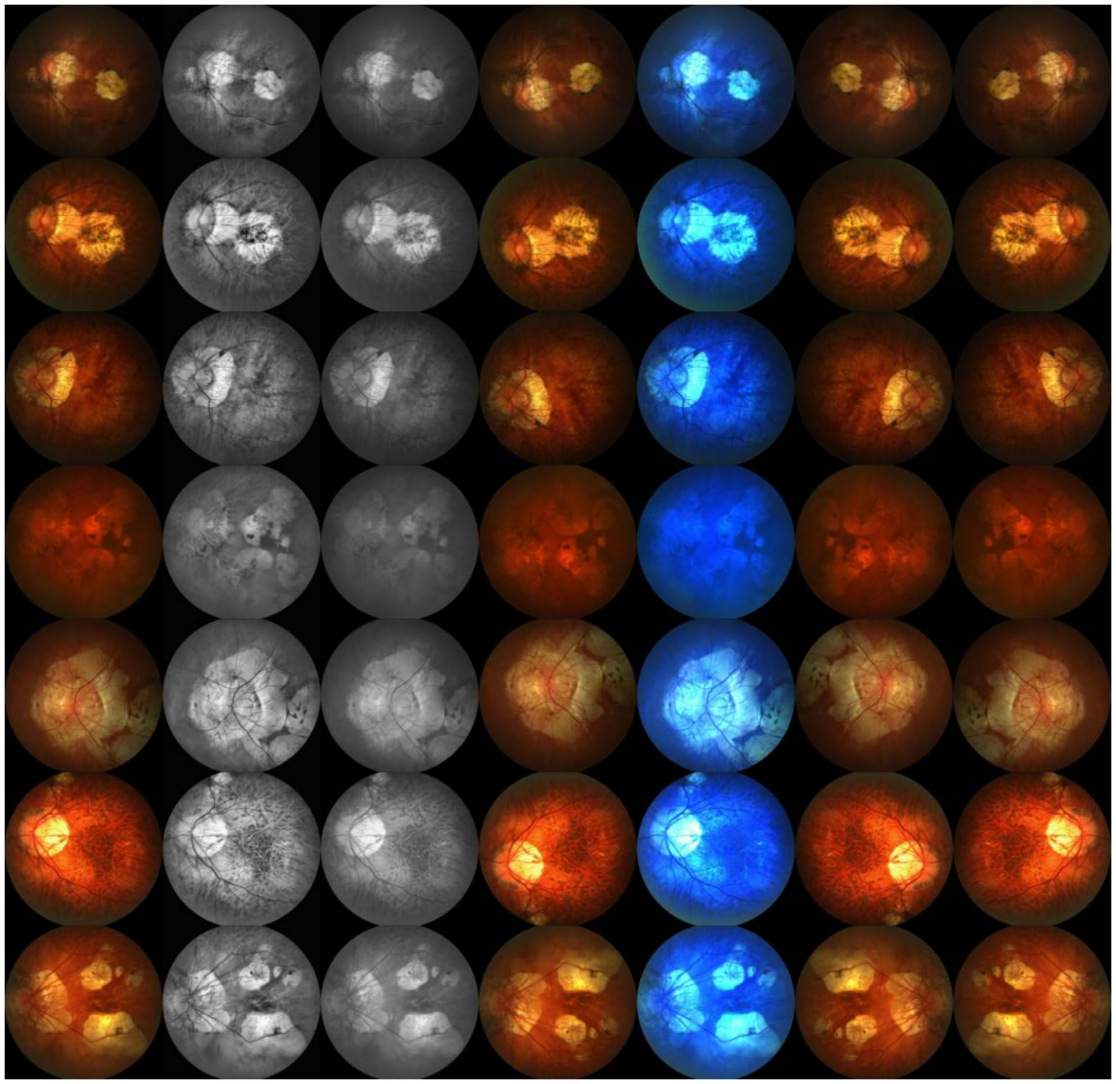
Sample display of the augmented dataset.

Due to the limited amount of data in the dataset, we divided the 795 images into a training set and a validation set in a 9:1 ratio, resulting in only 716 images in the training set and 79 images in the validation set. To increase the sample size of the training set, we applied various data augmentation techniques to the dataset, including histogram equalization, grayscale transformation, horizontal flipping, linear color transformation, rotation transformation, and vertical flipping. These data augmentation techniques expanded the total capacity of the dataset by 6 times, increasing the training set to 5,012 images and the validation set to 553 images. These data augmentation methods effectively increased the diversity of the training data, thereby improving the model’s generalization ability. A sample of the augmented dataset is shown in Figure 7.

## 5 Results

### 5.1 Comparison of SMLS-YOLO with the YOLOv8 family

**Figure 8:**
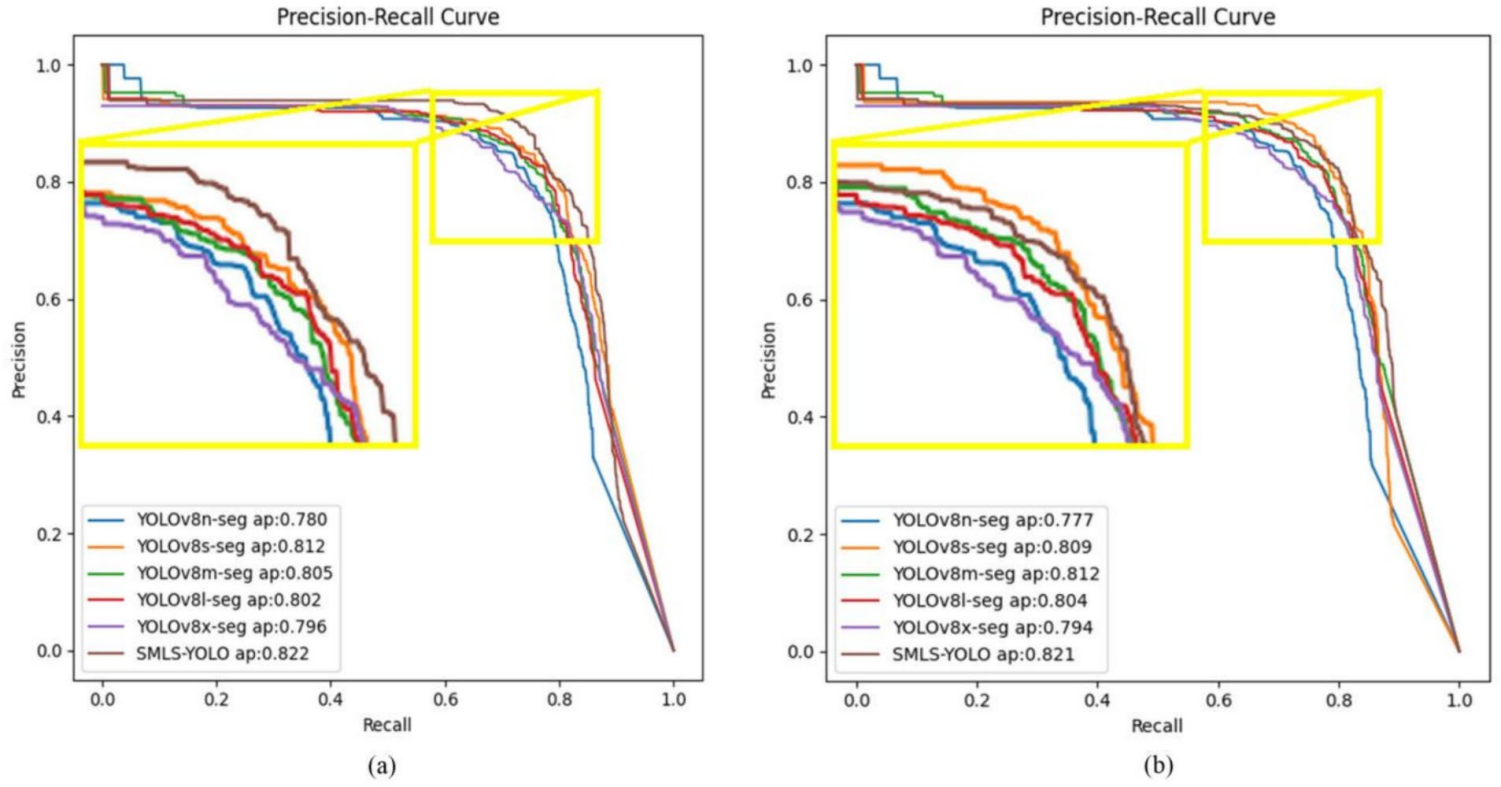
**(A)** Box P-R curve. **(B)** Mask P-R curve.

To demonstrate the superiority of SMLS-YOLO, we compared its performance with the YOLOv8 family on the augmented dataset. The results are shown in Table 2. In the YOLO instance segmentation experiments, the metrics include both Box and Mask components, corresponding to object detection and instance segmentation tasks, respectively. The object detection task focuses on locating and classifying target objects in the image, outputting the bounding box for each target object. These metrics reflect the model’s performance in object detection tasks. The instance segmentation task requires not only locating and classifying target objects but also predicting pixel-level segmentation masks for each target object. The Mask metrics reflect the model’s performance in instance segmentation tasks. From Table 2, we can see that on the augmented dataset, SMLS-YOLO achieved a precision of 89.2%, recall of 86.1%, mAP@0.5 of 89.0%, and F1 score of 88% for Box. For Mask, it achieved a precision of 89.9%, recall of 85.4%, mAP@0.5 of 88.9%, and F1 score of 88%. Compared to the baseline model YOLOv8n-seg, SMLS-YOLO improved the Box mAP@0.5 by 2.3% and the Mask mAP@0.5 by 3.9%. Additionally, SMLS-YOLO achieved a 46.7% reduction in model size, a 31.7% reduction in GFLOPS, and maintained nearly the same FPS. This indicates that SMLS-YOLO not only enhances detection and segmentation accuracy but also excels in computational efficiency and resource consumption. To visually demonstrate the performance of each model on the dataset, we plotted the P-R curves for the Atrophy class in both Box and Mask tasks. Figures 8A,B show the P-R curves for the Box and Mask tasks, respectively.

**Table 2:** Experimental results of SMLS-YOLO compared with YOLOv8 family.

Methods	Box				Mask				All		
	*p*	*R*	mAP@0.5	F1 score	*p*	*R*	mAP@0.5	F1 score	Params	GFLOPS	FPS
YOLOv8n-seg	89.7	83.2	86.7	86.0	89.4	82.8	85.9	86.0	3.26	12.0	93.3
YOLOv8s-seg	**91.3**	84.2	88.9	88.0	**90.5**	84.1	87.6	87.0	11.78	42.4	72.8
YOLOv8m-seg	88.8	85.5	88.6	87.0	89.0	85.2	88.4	87.0	27.22	110.0	61.5
YOLOv8l-seg	89.2	85.7	87.6	87.0	88.7	85.2	87.6	87.0	45.91	200.1	41.4
YOLOv8x-seg	85.4	86.6	87.7	86.0	86.3	85.2	87.1	86.0	71.72	343.7	26.4
SMLS-YOLO	89.2	86.1	**89.1**	**88.0**	89.9	**85.4**	**88.9**	**88.0**	**1.7**	**8.2**	92.8

Note: Bold values represent the best performance.

In the P-R curves shown in Figures 8A,B, SMLS-YOLO demonstrates significant advantages in both Box and Mask tasks. SMLS-YOLO maintains the highest precision across most recall levels, indicating higher accuracy in detecting and segmenting atrophic lesions, thereby reducing the risk of false positives and false negatives. In summary, SMLS-YOLO achieves comprehensive performance improvements in both Box and Mask tasks. Its overall performance surpasses that of the YOLOv8 family, proving the model’s comprehensive advantages in detection and segmentation tasks.

### 5.2 Comparison of SMLS-YOLO with advanced instance segmentation algorithms

To demonstrate that SMLS-YOLO has better generalization, we compare SMLS-YOLO, YOLOv5n-seg, YOLOv7-tiny-seg, YOLOv8n-seg, and YOLOv9’s Gelan-c-dseg, Gelan-c-seg, and YOLOv9-c-dseg, respectively, on the enhanced performance comparison on the dataset. The experimental results are shown in Table 3. Compared with other advanced target detection algorithms, SMLS-YOLO performs well on several key metrics.

**Table 3:** Experimental results of SMLS-YOLO compared with other advanced instance segmentation algorithms.

Methods	Box				Mask				All		
	*p*	R	mAP@0.5	F1 score	*p*	R	mAP@0.5	F1 score	Params	GFLOPS	FPS
YOLOv5n-seg	85.5	81.4	84.8	83.0	87.2	80.6	84.9	84.0	1.88	**6.7**	**111.8**
YOLOv7-tiny	**91.1**	78.3	83.9	83.0	**90.8**	79.8	84.2	84.0	6.99	47.7	101.2
YOLOv8n-seg	89.7	83.2	86.7	86.0	89.4	82.8	86.0	86.0	3.26	12.0	93.3
Gelan-c-seg	90.7	83.3	88.2	87.0	88.7	81.7	86.4	86.0	27.36	144.2	6.61
Gelan-c-dseg	88.6	84.4	87.9	86.0	88.4	83.4	87.0	86.0	27.39	145.2	5.72
YOLOv9-c-dseg	87.8	**84.6**	87.9	86.0	87.4	84.1	87.2	86.0	57.47	368.6	4.10
SMLS-YOLO	89.2	86.1	**89.1**	**88.0**	89.9	**85.4**	**88.9**	**88.0**	**1.7**	8.2	92.8

Note: Bold values represent the best performance.

In the Box task, SMLS-YOLO outperforms other models in several metrics, with mAP@0.5 reaching 89.1%, showing particularly outstanding performance. In contrast, the state-of-the-art Gelan-c-seg achieves an mAP@0.5 of 88.2%, which does not perform as well as SMLS-YOLO. Additionally, although the precision rate of YOLOv7-tiny reaches 91.1%, its recall rate is only 78.3%, leading to its lower overall performance, with an mAP@0.5 of 83.9%.

In the Mask task, SMLS-YOLO again leads in multiple metrics, further confirming its superiority. Additionally, SMLS-YOLO excels in model parameter count and computational efficiency. Its model parameters are only 1.7 M, significantly lower than those of other models. Furthermore, SMLS-YOLO’s GFLOPS is 8.2, and its FPS reaches 92.8, demonstrating high computational efficiency and real-time performance.

Precision (P) and Recall (R) are two key metrics used to evaluate model performance. Precision measures the accuracy of the model’s predictions, while Recall assesses the model’s ability to capture all relevant instances. Typically, there is a trade-off between Precision and Recall: increasing Precision by being stricter with positive class predictions (reducing false positives, FP) can lead to missing some true positives (increasing false negatives, FN), which in turn decreases Recall. Conversely, being more lenient with positive class predictions can increase Recall but May also result in more false positives, thus decreasing Precision. The mean Average Precision at Intersection over Union (IoU) threshold of 0.5 (mAP@0.5) metric balances different combinations of Precision and Recall to maximize the model’s overall performance. It calculates the average Precision and Recall across various thresholds, providing a comprehensive performance indicator by averaging these values. Therefore, even when there is a trade-off between Precision and Recall, mAP@0.5 offers a more holistic assessment of model performance. Compared to YOLOv8s-seg, SMLS-YOLO exhibits a slightly lower Precision but improved Recall and mAP@0.5, suggesting an overall enhancement in performance. Specifically, as shown in Table 2, SMLS-YOLO has a lower Precision (P) than YOLOv8s-seg, and in Table 3, SMLS-YOLO has a lower Precision (P) than YOLOv7-tiny. However, when considering the mAP@0.5 metric, which measures overall performance, SMLS-YOLO outperforms both YOLOv8s-seg and YOLOv7-tiny. Additionally, our SMLS-YOLO is more lightweight than YOLOv8s-seg and YOLOv7-tiny, with GFLOPS being only 17% of that of YOLOv8s-seg and YOLOv7-tiny.

**Figure 9:**
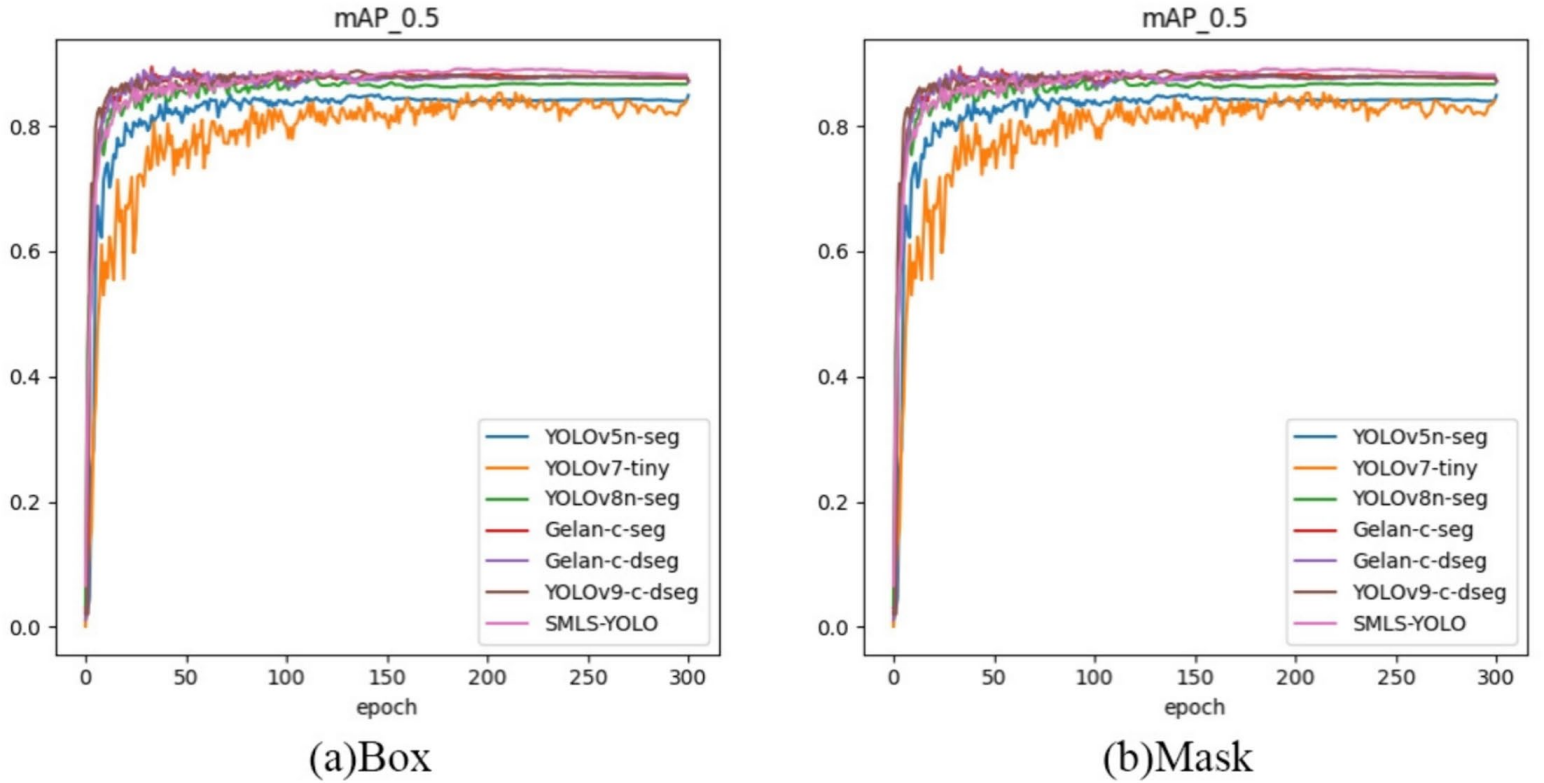
**(A)** Box mAP@0.5 curve. **(B)** Mask mAP@0.5 curve.

**Figure 10:**
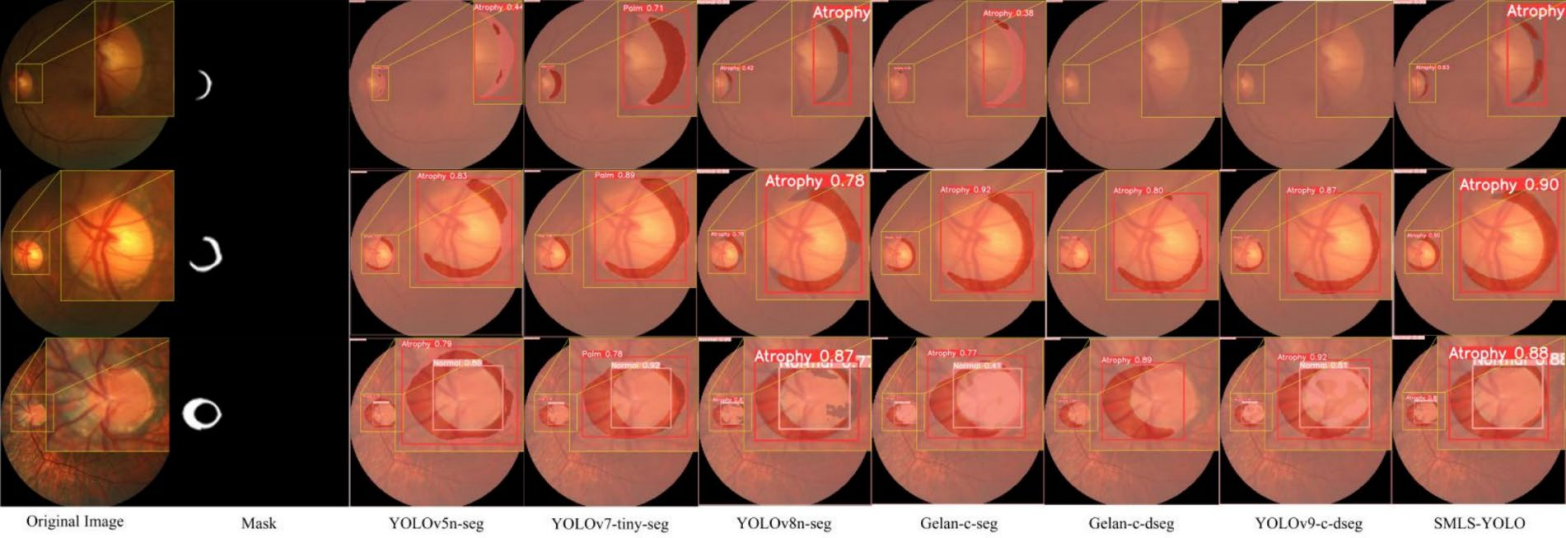
Visualization of SMLS-YOLO and other advanced instance segmentation algorithms results.

In summary, SMLS-YOLO not only excels in the Box task but also performs outstandingly in the Mask task. It achieves the best performance across multiple key metrics, demonstrating comprehensive advantages in both detection and segmentation tasks. Figures 9A,B show the mAP@0.5 curves for Box and Mask during the training process of seven networks. From these figures, it can be seen that SMLS-YOLO’s curve rises rapidly in the early stages of training, demonstrating its fast convergence ability. Additionally, its mAP@0.5 performance remains very stable and higher than other models throughout the training process, reflecting its stability and consistency. This indicates that SMLS-YOLO not only converges quickly in the early stages but also maintains high performance with minimal fluctuations throughout the training process, exhibiting excellent robustness and consistency. Furthermore, we visualized the detection results of the seven algorithms on the dataset to demonstrate SMLS-YOLO’s advantages over other advanced algorithms. Figure 10 shows the visualization results of the seven algorithms, where it can be seen that SMLS-YOLO achieves the best detection accuracy and prediction probability.

### 5.3 Comparison of SMLS-YOLO with classical segmentation networks

In order to verify the advantages and application potential of SMLS-YOLO, this paper compares the performance of SMLS-YOLO with classic segmentation algorithms such as UNet and the DeepLab series on an enhanced fundus color photography dataset. Table 4 presents the specific performance of SMLS-YOLO and these classic algorithms in terms of IoU, precision, recall, and F1-score.

**Table 4:** Experimental results of SMLS-YOLO compared with classical segmentation networks.

Methods	*p*	R	IoU	F1-score	Params	FPS
Unet	79.7	72.5	61.0	72.7	40.0	16.7
DeepLabV1	84.0	73.9	64.7	76.1	20.5	33.3
DeepLabV2	88.4	77.4	70.0	80.3	44.0	17.5
DeepLabV3	87.1	75.3	67.3	77.4	11.0	22.0
YOLOv8-seg	89.4	82.8	75.1	86.0	3.26	93.3
SMLS-YOLO	89.1	**88.9**	**76.6**	**88.0**	**1.70**	**92.8**

Note: Bold values represent the best performance.

Although UNet and the DeepLab series models are primarily used for semantic segmentation tasks, while SMLS-YOLO focuses on instance segmentation, the experimental results on the same dataset indicate that SMLS-YOLO not only surpasses these traditional semantic segmentation models in key performance indicators such as precision, recall, IoU, and F1-score, but also significantly reduces the number of parameters and increases processing speed. This suggests that, despite the differences in application domains, SMLS-YOLO still demonstrates strong generalization capabilities and superior performance when faced with semantic segmentation tasks.

**Figure 11:**
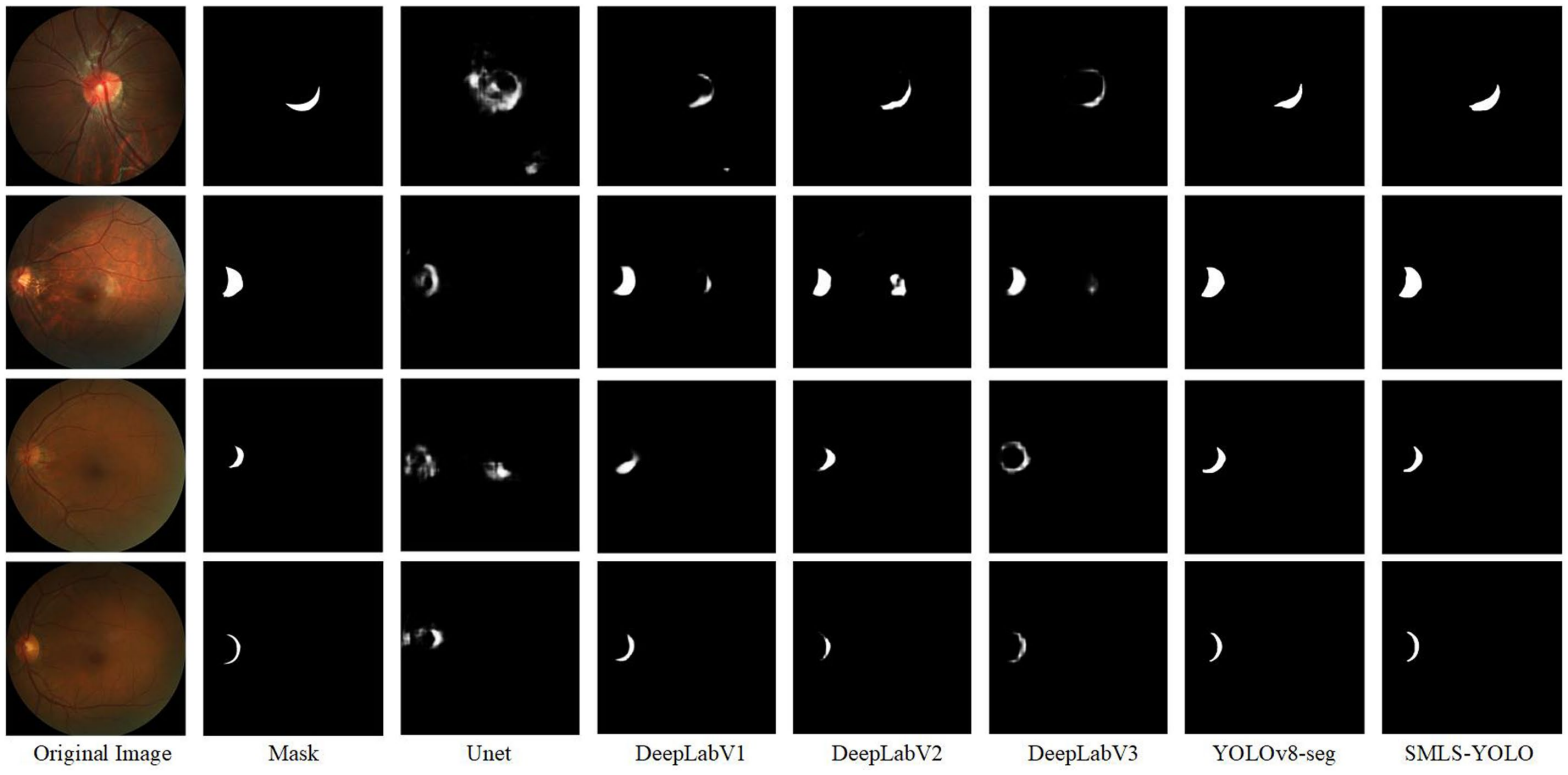
Visualization results of SMLS-YOLO compared with classical segmentation networks.

To validate the advantages of the SMLS-YOLO model across various performance metrics, this paper visualizes the detection results of SMLS-YOLO compared to classical segmentation networks, as shown in Figure 11. SMLS-YOLO demonstrates higher recognition accuracy and stronger adaptability when processing lesion areas, showing significant advantages.

### 5.4 Analysis of strategy effectiveness

To demonstrate the effectiveness of each improvement in SMLS-YOLO, we conducted an ablation study on the fundus image dataset. The results of the ablation study are shown in Table 5. Table 5 presents the detection performance achieved by the baseline algorithm YOLOv8n-seg with different combinations of components. It can be observed that each improvement strategy enhances the detection accuracy of the baseline algorithm to some extent.

**Table 5:** Experimental results under different improvement strategies.

Methods	StarNet	C2f-Star	Segment_LS	MHSA	mAP@0.5(Box)	mAP@0.5(Mask)	Params	Gflops	FPS
YOLOv8n-seg	–	–	–		86.7	86.0	3.26	12.0	93.3
A	√				87.6	86.5	2.47	10.4	96.7
B	√	√			87.7	86.4	2.27	10.0	97.7
C	√	√	√		87.8	86.3	1.50	8.1	95.3
D	√	√	–	√	89.1	87.7	2.46	10.1	94.1
E	√	–	–	√	88.6	87.1	2.66	10.5	93.8
F	√	–	√	–	88.4	86.9	1.70	8.4	96.5
G	√	–	√	√	88.9	88.5	1.90	8.6	94.4
SMLS-YOLO	√	√	√	√	89.1	88.9	1.70	8.2	92.8

From the aforementioned tables, it can be observed that the StarNet module has demonstrated excellent performance across multiple experiments. It not only effectively reduced the model’s parameter count and computational load but also improved detection accuracy to some extent. For instance, in experiments A, B, and C, despite the reduction in parameter count, the values of mAP@0.5(Box) and mAP@0.5(Mask) increased to varying degrees, indicating the module’s enhancement effect on model performance. By integrating StarNet as the Backbone of SMLS-YOLO, the baseline algorithm’s mAP@0.5 (Box) and mAP@0.5 (Mask) were, respectively, improved to 87.6 and 86.5%, while the model parameter count was reduced to 2.47 M. After incorporating the C2f-Star component, the mAP@0.5 (Box) further increased to 87.7%, the mAP@0.5 (Mask) slightly decreased to 86.4%, and the parameter count was reduced to 2.27 M. The introduction of the Segment_LS segmentation head further optimized the model’s balance, allowing the model to maintain low computational load while still improving detection accuracy. Additionally, the incorporation of the MHSA attention mechanism, although leading to a slight increase in parameter count, significantly enhanced model performance. In experiments E, F, and G, it proved the value of the MHSA module in improving model performance. Ultimately, after integrating all the improvement strategies, SMLS-YOLO’s mAP@0.5 (Box) and mAP@0.5 (Mask) were, respectively, increased to 89.1 and 88.9%, which is 2.4 and 3.9% higher than the baseline algorithm, with the parameter count being only 52% of YOLOv8n-seg.

**Figure 12:**
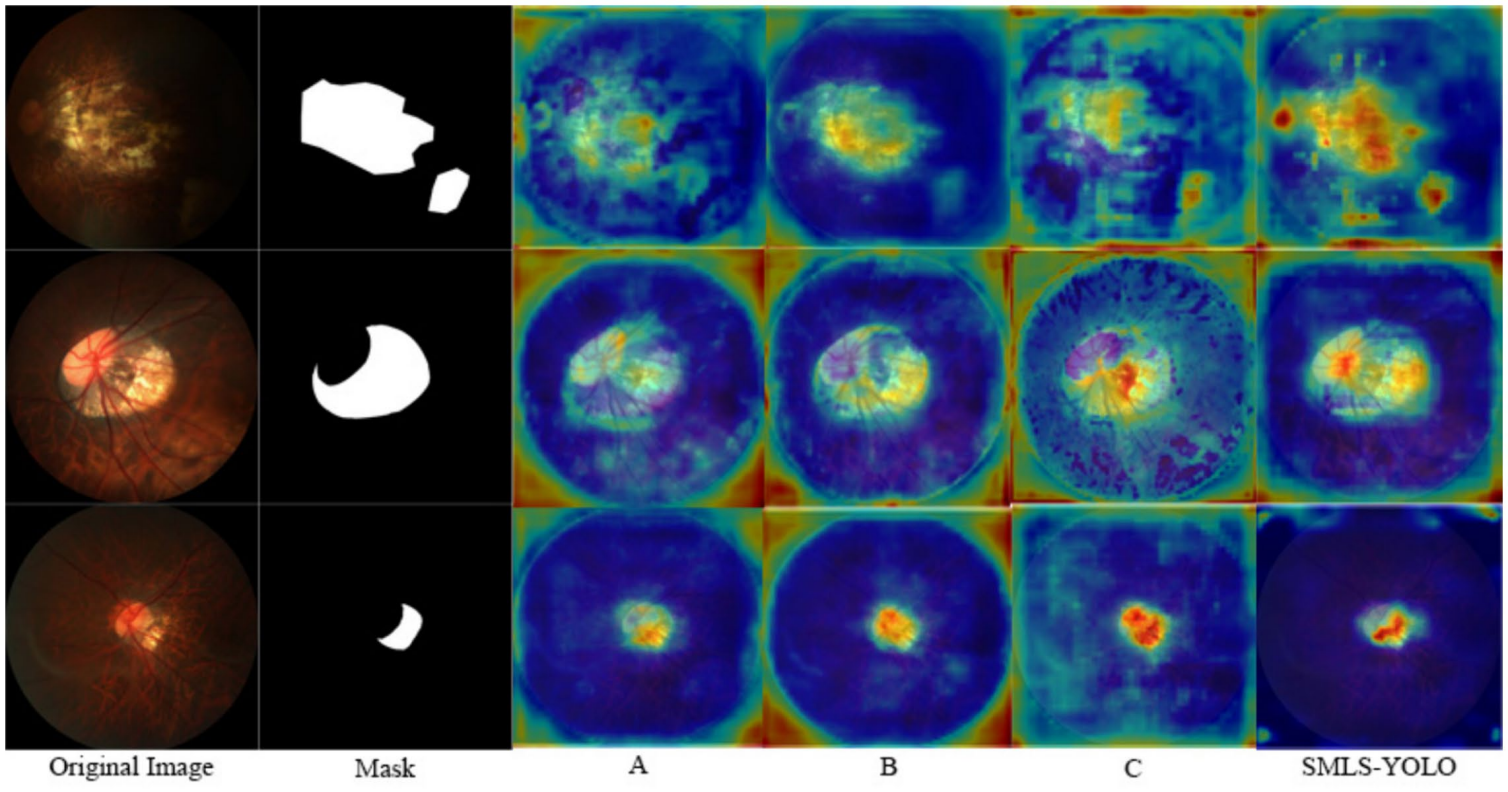
Heatmap results under different improvement strategies.

To further demonstrate the effectiveness of each improvement strategy, we conducted a heatmap visualization analysis of the model under various combinations of improvement strategies. Figure 12 shows the heatmap results under different combinations of improvement strategies. Through these visualizations, the performance enhancement effects of different improvement strategies on the model can be observed intuitively, thereby more clearly verifying the effectiveness of each improvement strategy.

## 6 Summary

In this paper, we proposed a novel instance segmentation algorithm named SMLS-YOLO, designed to tackle the challenges in detecting pathological myopia. Firstly, we introduced StarNet as the backbone network to efficiently extract feature information from images. Following this, we proposed a new feature extraction module, C2f-Star, which aims to more effectively integrate multi-level feature information produced by the backbone network, thereby enhancing performance while reducing the model’s complexity. Subsequently, to mitigate the issue of the original segmentation head’s large number of parameters, we proposed a new lightweight segmentation head, Segment_LS. This head leverages shared convolution and introduces scale adjustment operations, significantly reducing the computational burden during segmentation. Our Segment_LS segmentation head abandons the shared prototype masks of YOLOv8, thereby overcoming the segmentation head’s inherent limitations. As a result, our segmentation head does not require a large number of parameters to improve accuracy, thus significantly reducing the overall network parameters. Additionally, we integrated the Multi-Head Self-Attention (MHSA) mechanism to bolster the model’s capability to capture essential information in images, thereby improving the overall performance of SMLS-YOLO. Experiments conducted on fundus images dataset with pathological myopia demonstrate that SMLS-YOLO achieves advanced performance. Looking ahead, we intend to explore model pruning and knowledge distillation techniques to further refine the model’s efficiency and develop even more lightweight algorithms.

## Data Availability

The original contributions presented in the study are included in the article/supplementary material, further inquiries can be directed to the corresponding author.
